# Aneurysmal subarachnoid hemorrhage-risk score—impact of pre-existing cardiovascular risk factors on functional patient outcomes

**DOI:** 10.3389/fneur.2026.1781480

**Published:** 2026-03-11

**Authors:** Helen Ritter, Dirk Halama, Felix Arlt, Anika Stockert, Robert Werdehausen, Karl-Titus Hoffmann, Cindy Richter

**Affiliations:** 1 Department of Neuroradiology, Leipzig University Hospital, Leipzig, Germany; 2 Department of Oral and Maxillofacial Surgery, Leipzig University Hospital, Leipzig, Germany; 3 Department of Neurosurgery, Leipzig University Hospital, Leipzig, Germany; 4 Department of Neurology, Leipzig University Hospital, Leipzig, Germany; 5 Department of Anaesthesiology and Intensive Care Medicine, Medical Faculty, Otto-von-Guericke University Magdeburg, Magdeburg, Germany

**Keywords:** aneurysmal subarachnoid hemorrhage, cardiovascular risk-factors, functional outcome, prediction model, risk score

## Abstract

**Background:**

Predictive tools for assessing outcomes after aneurysmal subarachnoid hemorrhage (aSAH) are limited, particularly with respect to long-term functional outcome. Reliable risk stratification in the early course of aSAH is crucial for determining optimal patient management, effective use of clinical resources, and ultimately improving patient outcomes. This study aimed to design a prognostic score based on retrospectively collected clinical variables to predict functional outcome or delayed cerebral ischemia as primary endpoints in patients with aSAH.

**Methods:**

Between January 2014 and March 2022, 386 patients with aSAH were admitted to our hospital. Two hundred thirty of these patients were included in our study. Seventeen clinical, radiological, and demographic variables were analyzed using the chi-squared test and logistic regression to identify significant predictors of an unfavorable outcome (mRS 4–6) after 6 months or DCI development. A nomogram defined the weighting of each factor within a newly developed aSAH-Risk score.

**Results:**

Significant risk factors were identified to predict functional outcome. Of these, five variables were included to create the aSAH-Risk score with a maximum of 13 points: arterial hypertension (*p* = 0.001, no = 0, yes = 2), intracranial vasosclerosis (*p* = 0.0002, none = 0, yes = 2), modified Fisher scale (*p* < 0.001, scale 1 = 0, scale 3 = 2, scale 2 or 4 = 3), intracerebral hemorrhage (no = 1, yes = 2) and World Federation of Neursosurgical Societies grading (*p* < 0.001, 1 = 0, 2 = 1, 3 or 4 = 3, 5 = 4). Forty percent was the minimal calculated risk of an unfavorable outcome for an aSAH patient, increasing to 80% with an aSAH-Risk score of 13 points. An external cohort is required to validate the proposed score for general applicability.

**Conclusion:**

The aSAH-Risk score is a novel clinical tool to identify patients in need of long-term daily life assistance at admission.

## 1 Introduction

Aneurysmal subarachnoid hemorrhage (aSAH) has an overall incidence of 7.9 (95% CI, 6.9–9.0) per 100,000 person-years (1). It mostly affects people at a younger age, when permanent disability has a particularly more substantial social and economic impact. One critical challenge in patients with aSAH is the occurrence of complications such as hydrocephalus and delayed cerebral ischemia (DCI), including cerebral vasospasm, which result in poorer functional outcomes (2, 3). Therefore, identifying patients who are more susceptible to complications helps to determine the utilization of clinical resources, such as the frequency of clinical and radiological monitoring. Traditionally, prognostic models such as the World Federation of Neurosurgical Societies (WFNS) grading scale (4) and Hunt and Hess (H&H) scale (5) are used, which allow for the distinction of the severity of aSAH primarily based on clinical appearance. The low specificity of the WFNS grading scale with 0.19, confirms its limited prediction (6). Recent approaches, such as the nomogram for predicting the risk of poor prognosis with poor-grade aSAH following microsurgical clipping by Zhou et al. (7) or the volumetrically-enhanced SAH (eSAH) score by Sharma et al. (8), have improved prognostication by integrating further factors. The weighted eSAH score, based on the Glasgow Coma Scale (GCS), age, and aSAH volume, was strongly predictive of outcome (8). Despite these advances, only the WFNS and the H&H scales are still mainly applied in clinical routine. Scores that cannot be easily obtained from existing clinical routine information, because they include special laboratory parameters and volumetric measurements, are of limited use for actual care. Table 1 summarizes the pre-existing prediction models (4, 5, 7–18). Even though cardiovascular risk factors are known to play a significant role in the development and rupture of aneurysms as well as the outcome after aSAH (19–22), they remain underrepresented in most existing scores. For example, arterial hypertension is only included in two of the shown preexisting scores, the Subarachnoid Hemorrhage International Trialists (SAHIT) score (16) and the Functional Recovery Expected after Subarachnoid Hemorrhage (FRESH) score (12), highlighting the need for further analysis and novel predictive models, that account for these parameters.

**Table 1:** Pre-existing prediction models.

Score	Author, Year	*n*	Variables	Prognostic value
BEHAVIOR score	Jabbarli et al., 2015 (9)	632	Blood on CT (Fisher ≥3) Patient age ≥55 years H&H ≥ 4 EVD Vasospasm (angiogram) ICP > 20 mmHg Treatment of multiple aneurysms	Cerebral infarction Functional outcome (mRS at discharge & 6 months) In-hospital mortality rates
Early DCI Score	Fang et al., 2019 (10)	702	WFNS Modified Fisher scale SEBES Score IVH (assessed within 72 h)	DCI
eSAH score	Sharma et al., 2023 (8)	277	SAH volume GCS Patient age	Outcome (mRS discharge) DCI In-hospital mortality
Fisher Scale	Fisher, 1980 (11)	47	Amount and distribution of SAH on CT	Development of cerebral vasospasm
FRESH score	Witsch et al., 2016 (12)	1,619	H&H APACHE 2-physiologic score Patient age Rebleed within 48 h	Long-term outcome (mRS at 6 months)
HAIR score	Lee et al., 2014 (13)	400	H&H Patient age Rebleed within 48 h	In-hospital mortality rate
Hunt and Hess score	Hunt and Hess, 1968 (5)	275	Level of consciousness Headache severity Meningeal signs Focal neurological deficits	Stratify surgical risk and expected survival
Modified Fisher Scale	Frontera et al., 2006 (14)	1,355	Subarachnoid blood volume Presence of IVH on CT	Development of cerebral vasospasm
Nomogram for poor-grade SAH	Zhou et al., 2023 (7)	150	Patient age H&H GCS Aneurysm size Refractory fever	Outcome (GOS 6 months)
SAFIRE score	Van Donkelaar et al., 2019 (15)	1,215	Patient age WFNS (after resuscitation) Aneurysm size Fisher Scale	Functional outcome at 2 months (mRS)
SAHIT score	Jaja et al., 2018 (16)	10,936	Patient age Premorbid hypertension WFNS at admission Fisher Scale Size and location of aneurysm Method of treatment	Functional outcome (GOS at 3 months) Mortality
SEBES score	Ahn et al., 2018 (17)	164	Sulcal effacement and GWM disruption on CT at two bilateral levels (insula, centrum semiovale)	DCI Functional outcome (mRS at 6 months)
VASOGRADE score	De Oliviera Manoel et al., 2015 (18)	746	Modified Fisher scale WFNS	DCI
WFNS grading system	World federation of neurosurgical societies, 1988 (4)	n.m.	GCS Focal neurological deficits	Estimate patient prognosis Standardized evaluation of patient treatment Quantifying a change in status over time

Scores/Scales: APACHE, Acute Physiology And Chronic Health Evaluation; BEHAVIOR, Blood on CT, Elderly, H&H, Acute Hydrocephalus, Vasospasm, ICP, Overtreatment, Risk; eSAH, early subarachnoid hemorrhage; FRESH, Functional Recovery Expected after Subarachnoid Hemorrhage; GCS, Glasgow Coma Scale; HAIR, H&H, Age, IVH, Re-bleeding; H&H, Hunt and Hess; mRS, modified Rankin scale; SAFIRE, Size of aneurysm, Age, Fisher grade, rWFNS (WFNS after resuscitation); SAHIT, Subarachnoid Hemorrhage International Trialists; SEBES, Subarachnoid Hemorrhage Early Brain Edema Score; VASOGRADE, proper noun; WFNS, World Federation of Neurosurgical Societies.

CT, Computer tomography; DCI, delayed cerebral ischemia; EVD, external ventricular drain; GOS, Glasgow Outcome Scale; GWM, gray-white matter; ICP, intracranial pressure; IVH, intraventricular hemorrhage; SAH, subarachnoid hemorrhage.

Our goal was to design a prognostic aSAH-Risk score based on readily available information that incorporates cardiovascular risk factors and morbidities, the severity of aSAH, and the clinical condition on admission. Functional outcome, as measured by the modified Rankin Scale (mRS), and the development of DCI served as primary endpoints.

## 2 Methods

### 2.1 Population/study design and recruitment

**Figure 1:**
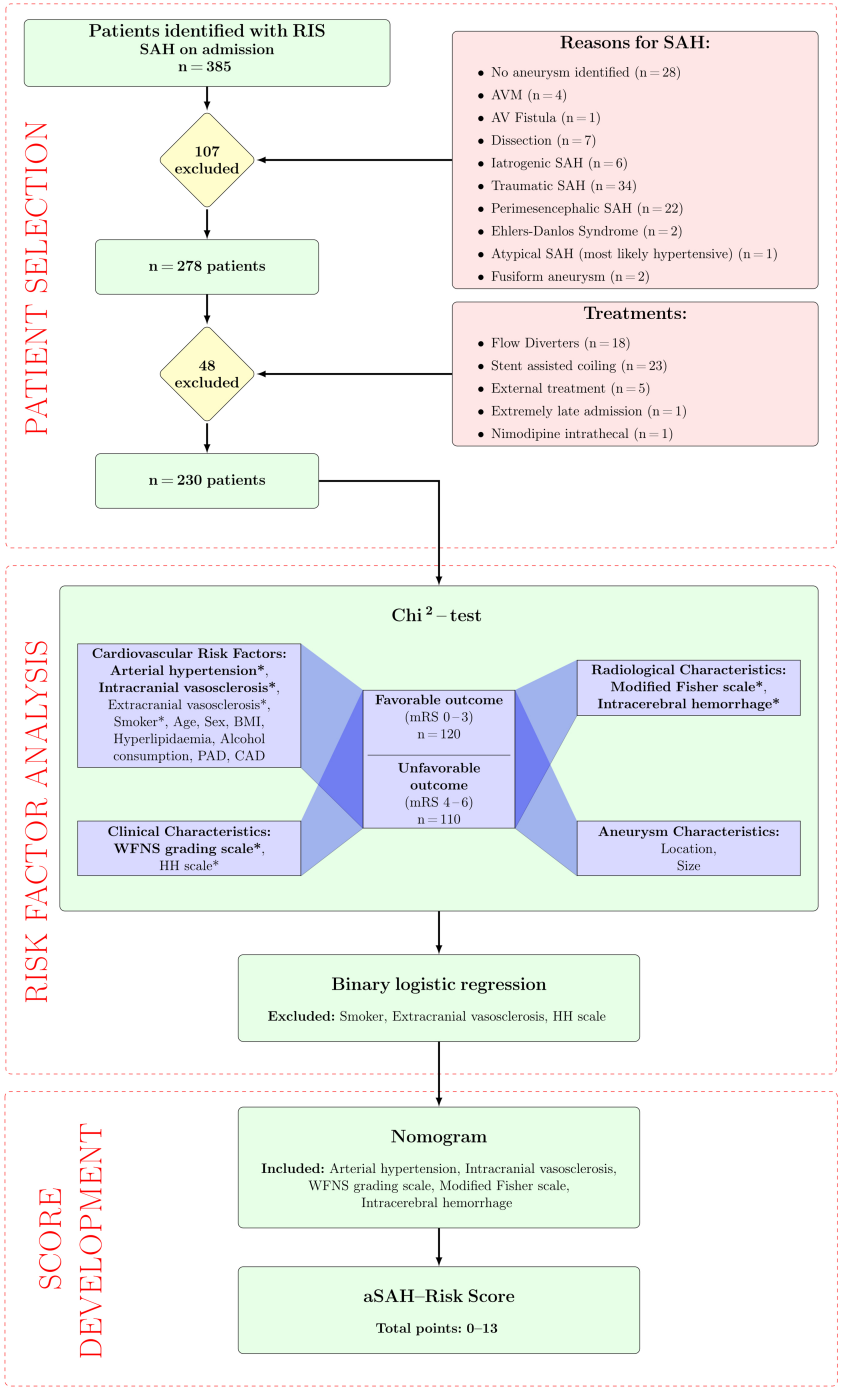
Flowchart. AV, arteriovenous; AVM, arteriovenous malformation; mRS, modified Rankin scale; RIS, radiology information system; SAH, subarachnoid hemorrhage.

We retrospectively screened our radiology information system (RIS) for patients with SAH admitted to our hospital between January 2014 and March 2022 (Figure 1). In total, 385 patients were identified, of whom 230 were included in our study. The local Ethics Committee approved this study (412/19-ek), and it was conducted in accordance with the ethical standards outlined in the Declaration of Helsinki. Inclusion criteria were an aSAH verified by computed tomography (CT), magnetic resonance imaging (MRI), or lumbar puncture. Patients with following conditions were excluded: no aneurysm identified (*n* = 28), arteriovenous malformations (AVM) (*n* = 4), arteriovenous fistulas (*n* = 1), dissections (*n* = 7), iatrogenic bleeding (*n* = 6), traumatic SAH (*n* = 34), perimesencephalic SAH without aneurysm (*n* = 22), Ehlers-Danlos Syndrome (*n* = 2), atypical bleeding (*n* = 1), fusiform aneurysm (*n* = 2), flow diverter implantation (*n* = 18), stent-assisted coiling (*n* = 23), external treatment (*n* = 5), extremely late admission (*n* = 1) or intrathecal nimodipine application (*n* = 1).

### 2.2 Data collection

Data collection was based on clinical and radiological findings according to established definitions. If a disease, addiction or medication was not mentioned in the patient’s medical records, it was rated as not present. The initial clinical presentation was assessed using both the H&H scale and the WFNS grading scale. The obtained demographic information and cardiovascular risk factors from the patient’s medical records are summarized in Table 2. Age and body mass index (BMI) were recorded as continuous variables and used in this manner in all statistical analyses. The presence of arterial hypertension was defined as the prescription of antihypertensive medication at the end of the in-hospital stay. Other cardiovascular risk factors, such as smoking, alcohol consumption, peripheral artery disease (PAD), and coronary artery disease (CAD) were extracted from patient records, including physician reports and discharge summaries. Smoking, alcohol consumption, and PAD were listed as dichotomous variables (yes/no). CAD was graded into none, one-vessel, two-vessel disease, or three-vessel disease based on the documentation in the medical records. No patients with three-vessel disease were identified. For hyperlipidemia, the highest measured triglyceride levels during the inpatient stay were assessed, as recorded in the morning clinical routine blood sampling. The values were included in the analysis as continuous variables.

**Table 2:** Demographics in correlation with clinical outcome.

Characteristics	Subdivisions	*n* (%)	*n* (%)	Chi^2^-test
		mRS 0–3 (*n* = 120)	mRS 4–6 (*n* = 110)	*p*
Patient characteristics				
Patient age (years)	Range (mean)	22–86 (49.6)	31–89 (58.9)	0.227
	<49	60 (26.1)	32 (13.9)	
	≥50	60 (26.1)	78 (33.9)	
Sex	Female (%)	32.6	29.6	1
BMI	<18.5	1 (0.4)	0 (0.0)	0.611
	18.5–24.9	54 (23.6)	40 (17.5)	
	≥25	64 (27.9)	70 (30.6)	
Risk factors				
Arterial hypertension	Yes	76 (34.9)	91 (41.7)	<0.001
	No	44 (20.2)	7 (3.2)	
Hyperlipidaemia	Yes	59 (25.7)	36 (15.7)	0.060
	No	60 (26.1)	64 (27.9)	
Smoker	Yes	37 (16.1)	18 (7.8)	0.015
	No	83 (36.1)	92 (40.0)	
Alcohol consumption	Yes	6 (2.6)	10 (4.3)	0.338
	No	114 (49.6)	100 (43.5)	
Peripheral artery disease	Yes	2 (0.9)	2 (0.9)	1
	No	118 (51.3)	111 (47.0)	
Coronary artery disease	none	119 (51.7)	107 (46.5)	0.463
	1 vessel	0 (0.0)	1 (0.4)	
	2 vessel	1 (0.4)	2 (0.9)	
Intracranial vasosclerosis	None	63 (27.4)	29 (12.6)	<0.001
	Mild	35 (15.2)	38 (16.5)	
	Moderate	12 (5.2)	18 (7.8)	
	Severe	10 (4.3)	25 (10.9)	
Extracranial vasosclerosis	None	53 (23.0)	34 (14.8)	0.001
	Mild	12 (5.2)	20 (8.7)	
	Moderate	3 (1.3)	11 (4.8)	
	Severe	0 (0.0)	6 (2.6)	
	Not available	91 (39.6)		
Clinical characteristics				
WFNS grading scale	1	47 (20.4)	10 (4.3)	<0.001
	2	33 (14.3)	11 (4.8)	
	3	10 (4.3)	13 (5.7)	
	4	12 (5.2)	26 (11.3)	
	5	18 (7.8)	50 (21.7)	
Hunt and Hess scale	1	29 (12.6)	4 (1.7)	<0.001
	2	46 (20.0)	14 (6.1)	
	3	15 (6.5)	24 (10.4)	
	4	15 (6.5)	25 (10.9)	
	5	15 (6.5)	43 (18.7)	
Modified Fisher scale	0/1	12 (5.3)	0 (0.0)	<0.001
	2	8 (3.5)	2 (0.9)	
	3	33 (14.5)	9 (4.0)	
	4	64 (28.2)	99 (43.6)	
Intracerebral hemorrhage	Yes	11 (4.8)	33 (14.3)	<0.001
	No	109 (47.4)	77 (33.5)	
Aneurysm location	ACA	59 (25.7)	45 (19.6)	0.261
	MCA	22 (9.6)	31 (13.5)	0.106
	ICA	28 (12.2)	21 (9.1)	0.533
	Posterior circulation	11 (4.8)	13 (5.7)	0.659
Aneurysm size	<10 mm	100 (43.9)	78 (34.2)	0.512
	≥10 mm	19 (8.3)	31 (13.6)	

ACA, anterior cerebral artery; BMI, body mass index; ICA, internal carotid artery; ICH, Intracerebral hemorrhage; MCA, middle cerebral artery; mRS, modified Rankin scale; WFNS, World Federation of Neurosurgical Societies.

### 2.3 Vasosclerosis

**Figure 2:**
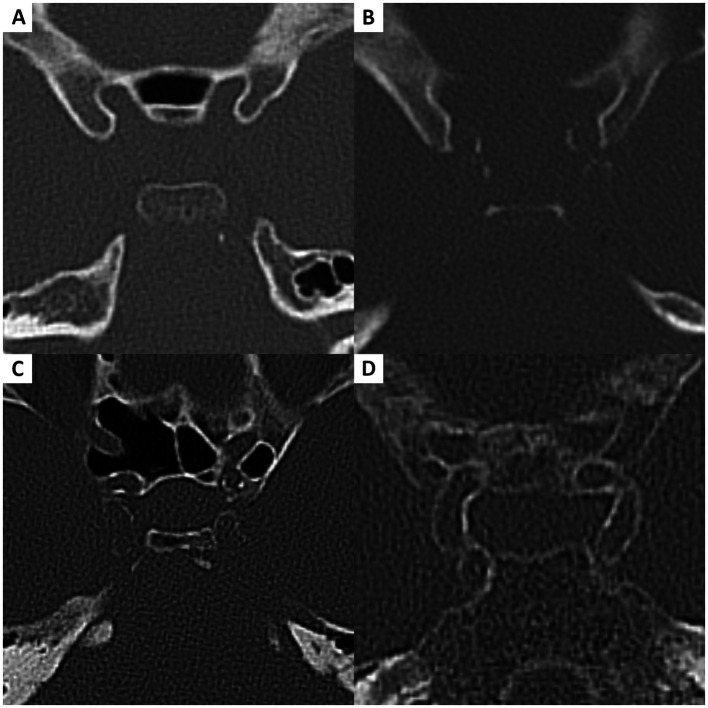
Vasosclerosis grading. **(A)** Grade 0 = no plaques visible. **(B)** Grade 1 = focal changes without circumferential configuration. **(C)** Grade 2 = beginning circumferential calcifications without segmental distribution. **(D)** Grade 3 = continuous circumferential calcifications with segmental distribution.

We classified the severity of intracranial vasosclerosis, from none to severe, using our newly developed vasosclerosis grading system (Figure 2). The score ranged from 0 to 3, with increasing intensity from none to severe. A score of zero meant that there were no plaques visible on the bone window CT scan (Figure 2A). Mild vasosclerosis (score = 1) was characterized by focal, early-stage changes in the vessel wall without a circumferential configuration (Figure 2B). Moderate vasosclerosis (score = 2) was defined as beginning circumferential calcifications without fully developed segmental distribution (Figure 2C), and severe vasosclerosis (score = 3) as continuous circular vessel wall involvement over an extended vascular segment (Figure 2D). Extracranial vasosclerosis was assessed at the level of the carotid artery bifurcation using the same grading system. If the CT angiography of a patient was not available, the extracranial vasosclerosis score was recorded as missing score (Table 2).

**Table 3:** The aSAH-risk score.

Risk factor	Points
Arterial hypertension	
No	0
Yes	2
Intracerebral hemorrhage	
No	0
Yes	2
Intracranial vasosclerosis	
No	0
Yes	2
Modified Fisher scale	
I	0
III	2
II or IV	3
WFNS grading scale	
1	0
2	1
3 or 4	3
5	4

ICH, intracranial hemorrhage; WFNS, World Federation of Neurosurgical Societies.

### 2.4 Severity of injury and location of aneurysm

Data regarding the severity of the injury was collected from the medical records using the determined modified Fisher scale, H&H scale, and WFNS grading scale by the respective treating physician. One experienced neuroradiologist (CR) retrospectively assessed intracerebral hemorrhage (ICH), aneurysm location, size, and site of the ruptured aneurysm in the plain CT and digital subtraction angiography (DSA) at admission.

### 2.5 Standard aSAH treatment protocol

Treatment of the ruptured aneurysm was either performed endovascularly or with surgical clipping, as soon as possible, within 72 h of onset. All patients were monitored in our neurointensive care unit. Nimodipine was given from the day of admission orally (6 × 60 mg/day) or via gastric tube. In accordance with the local standard operating procedure for SAH management, mean arterial blood pressure was consistently maintained above 80 mmHg to prevent reduced cerebral perfusion.

### 2.6 Endpoints

The first primary endpoint was the occurrence of DCI from day three to discharge. DCI was defined as cerebral infarction identified on CT or MRI after excluding procedure-related infarctions. Furthermore, our definition comprised the onset of focal neurological impairments documented in medical records – such as hemiparesis, aphasia, apraxia, hemianopia, or neglect – or a decrease in the level of consciousness that cannot be accounted for in any other way. For example, through status epilepticus/seizures, delirium, severe metabolic changes or sepsis, which have the potential to cause altered consciousness.

The functional outcome of the patients at 6 months after the onset of aSAH served as the second primary endpoint. It was measured using the modified Rankin scale (mRS) at 6 months. If follow up data at 6 months was unavailable the follow up after 3 months or at discharge was considered. For an intended dichotomous classification, an mRS score of 0–3 was considered a favorable outcome, while an mRS score of 4–6 was considered an unfavorable outcome. Categorization was based on clinically meaningful thresholds. This data was obtained retrospectively from the medical records of the follow-up examinations.

### 2.7 Statistical analysis

A two-stage statistical analysis was performed with SPSS version 29.0 (IBM Corporation, New York, USA) and R version 4.4.2 (The R Foundation for Statistical Computing, Vienna, Austria) to identify significant predictors of DCI and an unfavorable outcome. In the first step, we performed univariate analyses (χ^2^ tests) to identify candidate predictors of poor outcome or DCI. A two-tailed value of *p* < 0.05 was considered indicative of statistical significance. Only one of the two endpoints presented a sufficient number of statistically significant endpoints to allow for multivariable modeling. Therefore, the multivariable analysis and score development were solely based on functional outcome rather than DCI. In the second step, variables with *p* < 0.05 were entered into a multivariable generalized logistic regression model to estimate the predictive value of each significant risk factor.

The primary endpoint, unfavorable outcome (mRS 4–6), was chosen as the dependent (indicator) variable. The independent variables included cardiovascular risk factors, clinical characteristics on admission, radiological findings, and aneurysm characteristics. The corresponding probability of an unfavorable outcome ranged from 0 to 1. Based on the beta-coefficients from the multivariable logistic regression, a nomogram was constructed to weight the individual predictors.

## 3 Results

### 3.1 Study population

A total of 230 patients suffering aSAH were included in the study. The median age was 53 years (interquartile range 45–63), and 62.2% were female. At the time of admission, the clinical presentation was heterogeneous. While about half of the study population presented in a good clinical condition, 46.1% of patients had a WFNS grading of 4–5 and 42.6% a H&H scale of 4–5, representing poor clinical condition. Radiologically, most patients presented with a modified Fisher scale of 4 (71.8%), corresponding to a pronounced subarachnoid and intraventricular hemorrhage (IVH). Accompanying ICH was present in 19.1% of patients. The most common localization of ruptured aneurysms was the anterior cerebral artery (ACA, 45.3%), including the anterior communicating artery (ACoA, 39.6%), followed by the middle cerebral artery (MCA, 23.1%). The majority of treatments were endovascular (74.8%), while one-fifth (18.7%) underwent surgical clipping.

### 3.2 Delayed cerebral ischemia

Thirty percent of patients developed a DCI in the course of their clinical stay. The distribution of patients across individual cardiovascular risk factors varied from the distribution regarding functional outcome. There was no significant association between DCI and functional outcome in our study cohort. Only the WFNS grading scale (*p* = 0.022), the H&H scale (*p* = 0.039), and alcohol consumption (*p* = 0.031) were significantly associated with DCI. The patient characteristics in correlation with DCI development are summarized in Supplementary Table S1.

### 3.3 Functional outcome

More than half of patients (52.2%) had a favorable outcome, while the remaining patients (47.8%) had an unfavorable outcome. The overall mortality was 15.2%. In 70 patients, the 6-month mRS was not available, substantially affecting the group with a poor outcome (*n* = 65) at discharge.

A univariate analysis indicated that multiple factors were associated with an unfavorable outcome. Our study showed that patients with arterial hypertension were more likely to have an unfavorable outcome. Within the hypertension group (*n* = 167), 54.5% had a poor functional outcome, while in the non-hypertension group (*n* = 51) this proportion was only 13.7%. A similar observation was made for the radiological findings: the majority of patients had a modified Fisher scale grade of 4 (*n* = 163). Among these patients, 60.7% had an unfavorable outcome. Within the modified Fisher scale grade 0–2 group (*n* = 22) and the modified Fisher 3 group (*n* = 41), the minority (9.1 and 22%) experienced an unfavorable outcome. Furthermore, patients with radiological signs of intracranial vasosclerosis (*n* = 138) were associated with poor functional outcomes (58.9%), compared to 31.5% in the group without (*n* = 92). The presence of ICH at admission also proved to be a significant negative predictor. Seventy-five percent in the ICH group (*n* = 44) had an unfavorable outcome, compared to 41.2% in the non-ICH group (*n* = 186). Also, the clinical presentation, defined by H&H and WFNS scales, had an impact on the functional outcome. Focussing on the WFNS grading scale, 71.7% of patients with a WFNS grading of 4–5 (*n* = 106) had an unfavorable outcome, compared to 27.4% in the group with a WFNS grading of 1–3 (*n* = 124). Table 2 summarizes the patient characteristics in relation to the functional outcome.

### 3.4 Risk factors

In our study, multiple risk factors were identified as being associated with unfavorable outcomes. The most common risk factor was arterial hypertension, which 72.6% of patients presented with, followed by intracranial vasosclerosis (60.0%). Also, hyperlipidemia was present in 41.3% of patients, and 23.9% of patients were smokers. Of the available data, 37.4% of patients (52/139) had external vasosclerosis. Among the recorded risk factors, hypertension (*p* < 0.001), smoking (*p* = 0.015), intracranial vasosclerosis (*p* < 0.001), and extracranial vasosclerosis (*p* = 0.001) were significantly associated with poor clinical outcome. Hyperlipidemia showed a weak association that did not reach statistical significance (*p* = 0.060). No significant impact was observed for alcohol consumption, BMI, PAD, or CAD. Regarding clinical presentation at admission, patients with poor outcomes had significantly higher values on the WFNS grading scale, H&H scale, and modified Fisher scale (all *p* < 0.001). Additionally, the presence of ICH at admission significantly correlated with a poor functional outcome (*p* < 0.001).

### 3.5 Score development

The significant factors from the univariate analysis were grouped to exclude interference among the factors themselves as follows. One or a maximum of two significant factors per group were chosen for the scoring system.

- Cardiovascular risk factors: age, sex, BMI, arterial hypertension (included), hyperlipidemia, smoking, alcohol consumption, PAD, CAD, extracranial vasosclerosis, and intracranial vasosclerosis (included)
- Clinical characteristics on admission: H&H scale, WFNS grading scale (included)
- Radiological characteristics: modified Fisher scale (included), identified ICH (included)
- Aneurysm characteristics: location, size

Three significant predicative variables for functional outcome were excluded due to clinical considerations and collinearity: smoking, HH scale, and extracranial vasosclerosis. Smoking was excluded to prevent a misleading clinical interpretation, as smoking was significantly associated with favorable outcomes. This decision was made due to overall health concerns, as well as inconclusive evidence in the existing literature. Additionally, the HH scale was excluded because it and the WFNS grading scale record overlapping aspects of the initial clinical severity. To prevent collinearity, only one scale was included in our model. The WFNS was selected due to its superior objectivity and reproducibility. Finally, extracranial vasosclerosis was excluded due to a large number of missing cases (*n* = 91), which would have reduced the data quality. Moreover, by excluding extracranial vasosclerosis and only focusing on intracranial vasosclerosis, the risk of redundancy was reduced. Missing data were observed only in variables that were not statistically significant or were deliberately excluded due to limited data availability.

**Figure 3:**
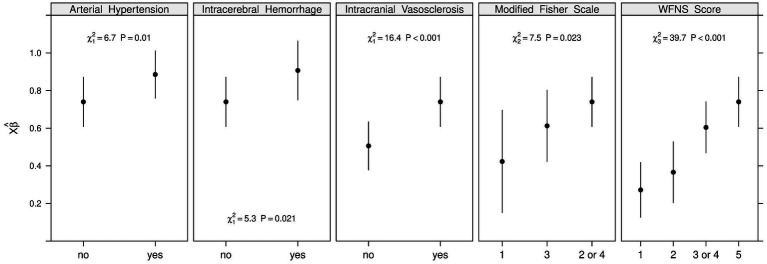
Impact of independent variables in the dichotomous linear regression model for unfavorable outcome. This figure displays the predicted probabilities of unfavorable outcomes, along with 95% confidence intervals for selected variables that demonstrated significant associations in prior Chi^2^ Tests (*p* < 0.05). Up to two variables were chosen to represent different domains: clinical characteristics on admission (WFNS grading scale), radiological characteristics (modified Fisher scale, ICH), and cardiovascular risk factors (arterial hypertension, intracranial vasosclerosis). All variables shown met the criteria for inclusion in the subsequent development of the nomogram. The coefficients (*β*) represent the change in the log-odds of the outcome for a one-unit increase in a predictor variable, holding other variables constant. Please note, that a grading of 3 on the modified Fisher scale was associated with a lower risk than the gradings 2 and 4, which both include IVH, in contrast to a grading of 3. Therefore, Fisher scale grades 2 and 4 were grouped. ICH, intracerebral hemorrhage; IVH, intraventricular hemorrhage; WFNS, World Federation of Neurosurgical Societies.

**Figure 4:**
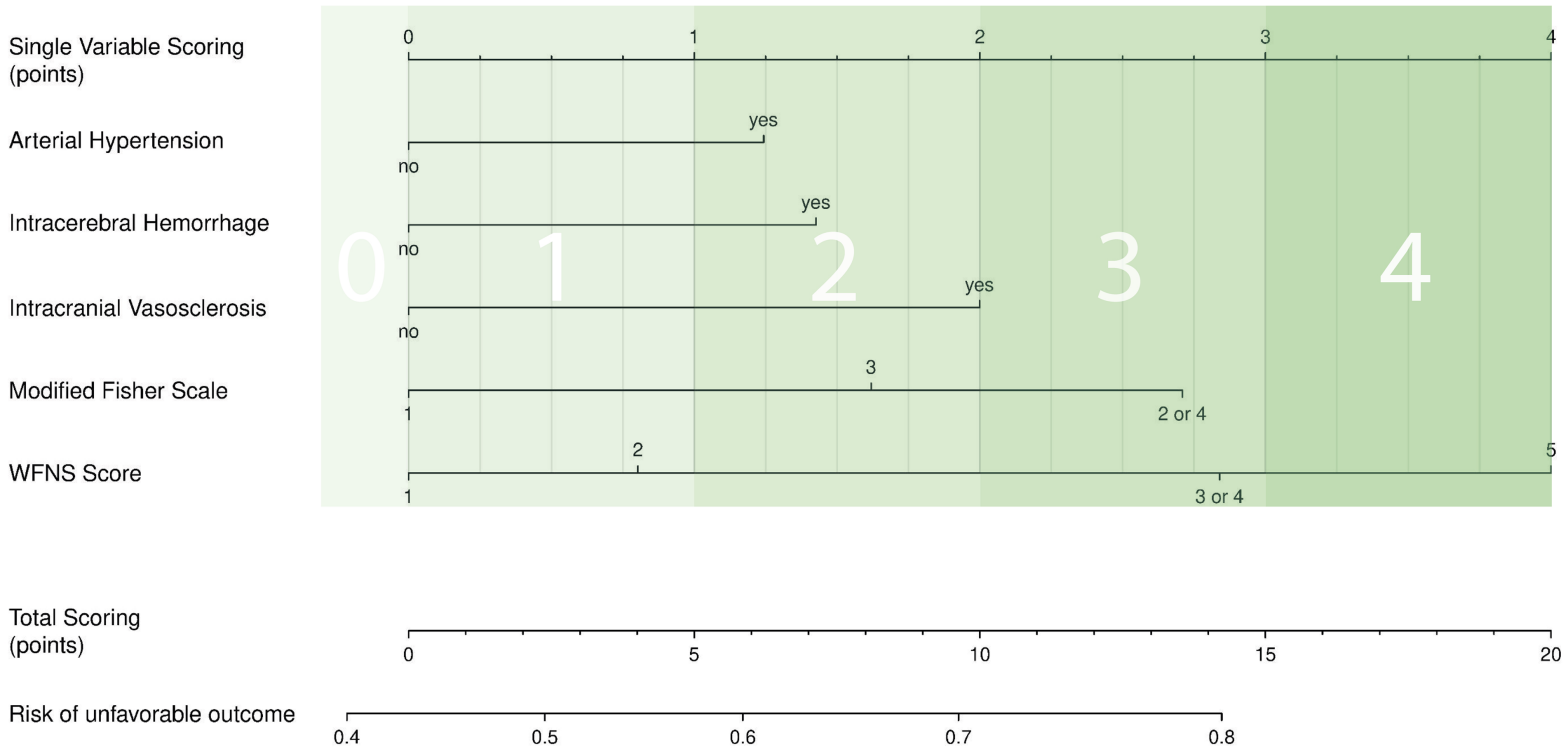
Nomogram to predict unfavorable outcome after aSAH, based on a multivariable logistic regression model. The variables identified as significantly predictive in the univariate analysis were included. Each predictor is assigned a score on a single-point scale proportional to its relative contribution in the model. They are arranged in descending order of importance for the model. The sum of all points represents the individual probability for an unfavorable outcome, which can be derived from the bottom scale. Thereby, the nomogram enables a patient-specific risk assessment ranging from 0.4 to 0.8 (Table 4). ICH, intracerebral hemorrhage; WFNS, World Federation of Neurosurgical Societies.

For point allocation, the value of each factor was defined using the dichotomous linear regression model (Figure 3) and a nomogram (Figure 4). For each variable, the corresponding point values were extracted and consistently rounded up to the following whole number to create a simplified point-based score. We included the WFNS grading scale as one of the most significant predictors, which therefore assigned up to 4 points, in a maximum of 13 possible points. Furthermore, the modified Fisher scale was an essential predictor, accounting for a maximum of 3 points. The presence of IVH (modified Fisher scale grade 2 or 4) on plane CT scans, as rated, outweighed the thickness of SAH (modified Fisher scale grade 3) in terms of correlation with poor functional outcomes. Therefore, Fisher scale grades 2 and 4 were grouped in the relevant table and figures. Finally, the presence of ICH, intracranial vasosclerosis and premorbid arterial hypertension were assigned a maximum of 2 points each. This allowed us to create a simple scoring system (Table 3), enabling the prediction of the risk of an unfavorable outcome after aSAH (Table 4).

**Table 4:** Risk of unfavorable outcome.

Risk of unfavorable outcome	Total points
0.4	0
0.5	1–3
0.6	4–7
0.7	8–12
0.8	13

## 4 Discussion

Although aSAH occurs less frequently compared to other subtypes of stroke, it is associated with disproportionately high morbidity and mortality. Aside from high mortality rates, survivors often suffer from persistent cognitive impairment as well as neuropsychiatric symptoms, such as anxiety and depression (23). These can affect daily functioning, including basic activities of daily living, return to work, and overall quality of life. Predicting functional outcomes can therefore assist in determining the utilization of clinical resources, such as the frequency of clinical and radiological monitoring. It can also inform neuro-rehabilitative treatment strategies, such as rehabilitation needs, timing, intensity and goals. Importantly, it can give a sense of direction for adequate education and preparation of family members, as well as counseling to support complex treatment decisions. Patients with aSAH are confronted with a complex spectrum of early and delayed complications. Early complications include rebleed, hydrocephalus, and seizures, while DCI is one of the most significant delayed complications (2). Primary brain injury initiates a complex pathophysiological cascade characterized by cerebral ischemia, inflammatory processes, and compromised blood–brain barrier integrity. These processes increase cerebral vulnerability to subsequent damaging events, such as cerebral vasospasm and microthrombosis, thereby facilitating the onset of DCI (3). DCI occurs in approximately 30% of patients, primarily between days 4 and 10 after SAH (24) and can progress to cerebral infarction, resulting in severe disability or death (3). Clinical deficits can occur in conjunction with angiographic evidence of vasospasm, but also independently of it (24).

In our analysis, we chose the functional outcome measured by mRS 6 months after onset of SAH as our primary endpoint, rather than DCI. The mRS is a validated and clinically accepted, patient-oriented measure of long-term functional outcome. In contrast, the exact definition and detection of DCI vary, allowing for fewer direct conclusions regarding long-term functional outcomes. Furthermore, we only found a limited statistically significant and consistent association between DCI and the cardiovascular risk factors investigated in our study. DCI, therefore, was not suitable as a predictable endpoint. We therefore decided to focus on developing a stable model for functional long-term outcome, which is practical and meaningful. The analyzed risk factors are discussed below.

### 4.1 Age

Age affects various stages of the clinical course in patients with intracranial aneurysms. While the evidence regarding the incidence of aneurysms, as well as the rupture rate in relation to age, varies (25, 26), age is an independent predictor of poor functional outcome after aSAH (27, 28). With increasing age, patients often have a more severe initial clinical presentation with impaired consciousness, dense subarachnoid clot formation, IVH, acute hydrocephalus, and a greater risk of rebleeding, all increasing the risk of unfavorable outcome (27). Furthermore, increasing age goes along with an increased risk of death after aSAH, with a hazard ratio of 6% per year of age (28). However, in our study cohort, no significant impact of age on unfavorable outcomes was found. This may be attributable to several factors, such as the small sample size, low variance of the predictor variable or other predictors masking the age effect.

### 4.2 Gender

Women are faced with a 1.3-times higher risk of aSAH, especially beyond the age of 55 years, when compared to men (1). The higher incidence of aneurysms and risk of rupture is suggested to be caused by a decrease in estrogen, accompanying menopause (29). However, this trend in increased aSAH rates does not necessarily correspond with increased rates of poor functional outcomes (30). Our findings do not indicate a significant relationship, which is in line with the majority of previous studies (3, 30), although one previous study suggested an association between gender and functional outcome (31).

### 4.3 BMI and hyperlipidemia

Multiple studies have investigated the correlation between BMI and functional outcome after aSAH, with heterogeneous results. Some have found no significant correlation (32, 33), supporting our findings. Another discussed the obesity paradox, where the overweight group showed an inverse relationship with a lower risk of death and poor outcomes, compared to the normal weight group (34). For future research, alternative markers, such as waist-to-hip ratio or inflammation-related biomarkers, could be analyzed, because BMI as a marker alone can only give a limited overview of metabolic burden. In our study, we included triglyceride levels as a component of the lipid profile, but found no significant correlation between increased triglyceride levels and functional outcome. The impact of triglycerides on the clinical course after aSAH is only partially illuminated, as most studies focus on total cholesterol and low-density lipoprotein levels (35) or the rupture risk (36) rather than outcomes. A study by Hou et al. investigated the relationship between the triglyceride-glucose index and functional outcome, finding a significant association with an increased risk of unfavorable outcomes (37). Due to the retrospective nature of our study, triglycerides were chosen as a parameter because the data were most readily available. However, the inability to determine lipid profile levels at standardized time points may have influenced the results, which should be taken into account for future research.

### 4.4 Arterial hypertension

Arterial hypertension is one of the most common modifiable risk factors in the general population. Our results, as well as those of most previous studies, support the inclusion of arterial hypertension as a predictor in the aSAH-Risk score described here. It increases susceptibility to vascular events by causing endothelial dysfunction and structural vascular damage. Non-modifiable risk factors for arterial hypertension include genetic predisposition and advancing age. However, there are many modifiable risk factors, such as obesity, lack of physical activity, smoking, high sodium intake, and excessive drinking (38). Furthermore, persistent arterial hypertension leads to left ventricular hypertrophy and diastolic dysfunction, vascular remodeling, and microangiopathy, which, among other things, increases the risk for atherosclerosis, myocardial infarction, ischemic and hemorrhagic stroke, aneurysm formation, and chronic kidney disease (38). Chronic arterial hypertension changes the fluid dynamics by inducing abnormal wall shear stress, leading to degenerative changes in the vascular wall, which increases the risk of aneurysm formation (39). Not only does uncontrolled arterial hypertension increase the risk of aneurysm formation, but it also increases the risk of aneurysm rupture, subsequently leading to aSAH (40). Preexisting arterial hypertension leads to an increased initial bleeding severity and a higher risk of rebleeding, both of which can lead to unfavorable outcomes (19). The SAHIT repository (20), an extensive pooled analysis of six studies with 7,249 patients, evaluated the prognostic value of premorbid hypertension, concluding that it was independently but weakly associated with poor outcome, with an adjusted OR of 1.38 (95% CI 1.25–1.53). The impact of premorbid hypertension likely reflects multiple mechanisms, including severity of initial hemorrhage, higher incidence of comorbidities, and higher likelihood of secondary neurological injury (20). Furthermore, it is associated with an elevated risk of cerebral infarction after aSAH, which likewise increases the risk of an unfavorable outcome (41). In our retrospective cohort, arterial hypertension was defined by the presence of discharge medication. Future score applications should rely on premorbid hypertension, defined by the presence of blood pressure medication at admission, to enable early prediction of functional outcomes. Due to the retrospective nature of this study, accurate determination of premorbid hypertension is challenging due to incomplete documentation and the confounding effects of acute illness on admission blood pressures. Using the prescription of antihypertensive medication at discharge, therefore, served as a surrogate for premorbid hypertension, as it portrays a longitudinal clinical assessment of the inpatient course, rather than a single acute measurement. While the timing of this assessment is a limitation, the observed association between hypertension and outcome is consistent across studies, supporting the clinical relevance of this variable.

### 4.5 Intracerebral and intraventricular hemorrhage

Another critical predictor of unfavorable outcome in our study population was the presence of an ICH at admission. Previous studies supported these findings (21, 42). This may be explained by the direct damage to intracranial parenchyma, as well as secondary effects such as increased intracranial pressure, development of brain edema, a higher rate of seizures, and the development of DCI (43). With terms of applicability in clinical practice, ICH was included in the aSAH-Risk score as a dichotomous variable (present/not present), not as a volume. Our study cohort revealed that patients with a modified Fisher scale of 2 or 4 tended to have worse functional outcomes compared to those with a modified Fisher scale of 3. This seems counterintuitive, as a modified Fisher scale of 3 represents a more prominent SAH than a modified Fisher scale 2, but no IVH. This suggests that the presence of IVH has a more significant impact on unfavorable outcomes compared to the amount of blood in the subarachnoid space alone. This observation is supported by previous studies, which have shown a significantly increased risk of unfavorable outcomes in the presence of IVH (44), as well as our findings. This can be explained pathophysiologically through the disruption of cerebrospinal fluid circulation, increased intracranial pressure, and inflammatory secondary processes (45).

### 4.6 Smoking

Contrary to common expectations, our data revealed a significant relationship between smoking and a favorable outcome (mRS 0–3 at 6 months after SAH) in the univariate analysis, but was excluded for multivariate analysis. The impact of smoking on clinical outcomes after aSAH remains controversial. Some studies indeed report a protective effect (46, 47) while another observed no significant effect (48). Dasenbrock et al. (47) confirmed that smoking was associated with better outcomes, even though smokers present with a higher number of comorbidities. Additionally, Krishnamurthy et al. (49) described smoking being associated with delayed neurological deterioration, albeit without evidence of a corresponding decline in clinical outcome, highlighting the complexity of the topic.

Furthermore, critical clinical considerations argue against including smoking in our prediction model, such as the fact that smoking is a risk factor for the development and rupture of cerebral aneurysms (40, 47). Therefore, despite statistical significance in the univariate analysis, we deliberately excluded smoking from the multivariate analysis for the development of the aSAH-Risk score based on several considerations, including conflicting evidence in the existing literature and overall health concerns. Our decision aims to prevent potential misunderstanding about the impact of smoking in SAH patients.

### 4.7 Vasosclerosis

We focused on intracranial vasosclerosis and omitted extracranial vasosclerosis from our analysis, as it would have led to unnecessary complexity and a partial lack of data. Data on extracranial vasosclerosis were only available for 139 of 230 patients (60.4%), because not all patients received a CTA, or the segment of interest was not visualized in imaging. Patients with high-grade extracranial or intracranial stenoses were excluded from the study.

Our developed prediction model for unfavorable outcomes builds upon existing models by integrating a broader range of risk factors that have not been included in pre-existing models. In addition to classical parameters, such as the WFNS grading scale, radiological factors, including the modified Fisher scale and ICH, are also taken into consideration. Furthermore, pre-existing cardiovascular risk factors such as arterial hypertension and vasosclerosis were integrated, as they have been discussed to increase the risk of poor outcomes (21, 50). The comprehensive integration of clinical, radiological, and pre-existing factors facilitates a more accurate and nuanced prediction of unfavorable outcome risk.

## 5 Limitations

This retrospective study with a focus on cardiovascular risk factors has several limitations. First, the limited sample size of the group does not allow for a more rigorous statistical analysis.

The retrospective nature of this study limited the assessment of cardiovascular risk factors and DCI, as it carries a risk of incomplete or inaccurate data on patient characteristics. The developed score was designed to capture premorbid arterial hypertension as a chronic vascular risk factor for an unfavorable outcome. Due to the retrospective nature of this study and incomplete documentation at discharge, premorbid hypertension needed to be assessed with the aid of antihypertensive medication prescribed at discharge. While this approach provides a longitudinal assessment of the inpatient course, misclassification cannot be excluded, as antihypertensive medication might have been initiated in patients with transient blood pressure increases, whereas patients with mild or untreated arterial hypertension may be underrepresented. Importantly, the score is intended for future use with directly assessed premorbid arterial hypertension and should be validated in an independent patient cohort.

Likewise, Triglyceride levels are not included in the emergency laboratory. Triglyceride levels are highly variable and should be interpreted with caution. Therefore, excluding these values from our risk model was optimal.

A further limitation concerns the primary endpoint: the assessment of functional outcome using the mRS at 6 months. The mRS at discharge was used for a subset of patients for whom mRS at 6 months was unavailable, either because of death at discharge (*n* = 39) or loss to follow-up for unknown reasons (*n* = 31). Discharge mRS may not fully reflect long-term functional outcomes, as neurological recovery or deterioration may occur and is not accounted for. Death (mRS 6) is an unchangeable condition that persists after 6 months. Patients with severe or moderately severe disabilities (mRS4-5) are not able to attend any follow-up appointments alone. The high level of effort accounts for the proportion of missing data among patients with unfavorable outcomes at discharge, supporting the persistence of poor condition. To define the aSAH-risk score, we analyzed outcome groups. Inner-group changes do not affect score accuracy.

Furthermore, although variations existed between the DCI group and the overall population, no statistical significance was established, which led to the exclusion of DCI from our predictive model. According to the definition by Vergouwen et al. (15), cerebral infarction on CT or MRI, along with clinical deterioration, is used to assess the development of DCI. Cerebral infarction was reliably evaluated radiologically in our data set. However, analyzing clinical deterioration requires thorough and consistent documentation, including a detailed description of infarction patterns and their relationship to particularly eloquent areas and systems, which is challenging given the retrospective nature of the study. This possibly accounts for the missing significance.

Another methodological limitation is that the study employed a single-center design, which restricts its generalizability. Differences in patient selection, treatment protocols, and resources can influence these results. Therefore, an independent dataset should be evaluated with the aSAH score in order to validate the predictive value.

## 6 Conclusion

In conclusion, our analysis established that high initial WFNS gradings and high grades in the modified Fisher scale, intracerebral hemorrhage, arterial hypertension, and intracranial vasosclerotic changes are associated with poor functional outcomes after aSAH. Due to its simplicity, the aSAH-Risk score is clinically feasible, especially in the acute stage of treatment. It enables the prediction of long-term risk for dependency on assistance in daily life, particularly for younger working-age patients, where permanent disability has a significant social and economic impact.

## Data Availability

The datasets presented in this study can be found in online repositories. The names of the repository/repositories and accession number(s) can be found at: https://doi.org/10.5281/zenodo.17339029.

## Abbreviations

ACA: Anterior cerebral artery
ACoA: Anterior communicating artery
aSAH: Aneurysmal subarachnoid hemorrhage
AVM: Arteriovenous malformation
BMI: Body mass index
CAD: Coronary artery disease
CI: Confidence interval
CT: Computed tomography
CTA: Computed tomography angiography
DCI: Delayed cerebral ischemia
GCS: Glasgow Coma Scale
H&H: Hunt and Hess
ICH: Intracerebral hemorrhage
ICP: Intracranial pressure
IQR: Interquartile range
IVH: Intraventricular hemorrhage
LDL: Low density lipid
MCA: Middle cerebral artery
MRI: Magnetic resonance imaging
mRS: Modified Rankin Scale
OR: Odds Ratio
PAD: Peripheral artery disease
RIS: Radiology information system
SAH: Subarachnoid hemorrhage
WFNS: World Federation of Neurosurgical Societies
