# Supplementary material for: Aneurysmal subarachnoid hemorrhage-risk score—impact of pre-existing cardiovascular risk factors on functional patient outcomes
Source: Front Neurol. 2026 Mar 11;17:1781480. doi: 10.3389/fneur.2026.1781480 (PMC13013078; doi:10.3389/fneur.2026.1781480)
Supplement: Supplementary file 1 [file Table_1.DOCX]

Supplementary Material

# Supplementary Table

**Table S1: Demographics in correlation with DCI development.**

Abbreviations: ACA= anterior cerebral artery, BMI= body mass index, ICA= internal carotid artery, ICH= Intracerebral hemorrhage, MCA= middle cerebral artery, mRS= modified Rankin Scale, WFNS= World Federation of Neurosurgical Societies

|  |  | **n(%)** | **n(%)** | **Chi^2^-test** |
| --- | --- | --- | --- | --- |
| Characteristics | Subdivisions | DCI (n=69) | No DCI (n=161) | p |
| **Patient Characteristics** | | | | |
| **Patient age (years)** | **Range (mean)** | 17-86 (53.0) | 7-89 (54.5) | 0.663 |
|  | <49 | 32 (13.9) | 60 (26.1) |  |
|  | ≥50 | 101 (43.9) | 37 (16.1) |  |
|  |  |  |  |  |
| **Sex** | **Female (%)** | 19.1 | 43.0 | 0.859 |
|  |  |  |  |  |
| **BMI** | <18,5 | 0 (0.0) | 1 (0.4) | 0.586 |
|  | 18,5-24,9 | 28 (12.2) | 66 (28.8) |  |
|  | ≥25 | 41 (17.9) | 93 (40.6) |  |
| **Risk Factors** | | | | |
| **Arterial Hypertension** | Yes | 54 (24.8) | 113 (51.8) | 0.451 |
|  | No | 13 (6.0) | 38 (17.4) |  |
|  |  |  |  |  |
| **Hyperlipidemia** | Yes | 46 (20.0) | 78 (33.9) | 0.330 |
|  | No | 23 (10.0) | 72 (31.3) |  |
|  |  |  |  |  |
| **Smoker** | Yes | 19 (8.3) | 36 (15.7) | 0.500 |
|  | No | 50 (21.7) | 125 (54.3) |  |
|  |  |  |  |  |
| **Alcohol consumption** | Yes | 9 (3.9) | 7 (3.0) | 0.031 |
|  | No | 60 (26.1) | 154 (67.0) |  |
|  |  |  |  |  |
| **Peripheral artery disease** | Yes | 1 (0.4) | 3 (1.3) | 1 |
|  | No | 68 (29.6) | 158 (68.7) |  |
|  |  |  |  |  |
| **Coronary artery disease** | none | 69 (30.0) | 157 (68.3) | 0.418 |
|  | 1 vessel | 0 (0.0) | 1 (0.4) |  |
|  | 2 vessel | 0 (0.0) | 3 (1.3) |  |
|  |  |  |  |  |
| **Intracranial vasosclerosis** | None | 28 (12.2) | 64 (27.8) | 0.368 |
|  | Mild | 21 (9.1) | 52 (22.6) |  |
|  | Moderate | 6 (2.6) | 24 (10.4) |  |
|  | Severe | 14 (6.1) | 21 (9.1) |  |
|  |  |  |  |  |
| **Extracranial vasosclerosis** | None | 23 (10.0) | 64 (27.8) | 0.744 |
|  | Mild | 11 (4.8) | 21 (9.1) |  |
|  | Moderate | 6 (2.6) | 8 (3.5) |  |
|  | Severe | 2 (0.9) | 4 (1.7) |  |

| **Clinical Characteristics** | | | | |
| --- | --- | --- | --- | --- |
| **WFNS grading scale** | 1 | 21 (9.1) | 36 (15.7) | 0.022 |
|  | 2 | 12 (5.2) | 32 (13.9) |  |
|  | 3 | 5 (2.2) | 18 (7.8) |  |
|  | 4 | 18 (7.8) | 20 (8.7) |  |
|  | 5 | 13 (5.7) | 55 (23.9) |  |
|  |  |  |  |  |
| **Hunt and Hess scale** | 1 | 10 (4.3) | 23 (10.0) | 0.039 |
|  | 2 | 18 (7.8) | 42 (18.3) |  |
|  | 3 | 15 (6.5) | 24 (10.4) |  |
|  | 4 | 17 (7.4) | 23 (10.0) |  |
|  | 5 | 9 (3.9) | 49 (21.3) |  |
|  |  |  |  |  |
| **Modified Fisher scale** | 0/1 | 3 (1.3) | 9 (4.0) | 0.440 |
|  | 2 | 1 (0.4) | 9 (4.0) |  |
|  | 3 | 15 (6.6) | 27 (11.9) |  |
|  | 4 | 49 (21.6) | 114 (50.2) |  |
|  |  |  |  |  |
| **Intracerebral hemorrhage** | Yes | 31 (13.5) | 13 (5.7) | 1 |
|  | No | 130 (56.5) | 56 (24.3) |  |
|  |  |  |  |  |
| **Aneurysm location** | ACA | 25 (10.9) | 79 (34.3) | 0.099 |
|  | MCA | 17 (7.4) | 36 (15.7) | 0.838 |
|  | ICA | 19 (8.3) | 30 (13.0) | 0.182 |
|  | Posterior circulation | 8 (3.5) | 16 (7.0) | 0.888 |
|  |  |  |  |  |
| **Aneurysm size** | <10mm | 123 (53.9) | 55 (24.1) | 0.525 |
|  | ≥10mm | 37 (16.2) | 13 (5.7) |  |
